# The Relationship of Rapid Eye Movement Sleep Behavior Disorder and Freezing of Gait in Parkinson’s Disease

**DOI:** 10.7759/cureus.12385

**Published:** 2020-12-30

**Authors:** Chelsea Mae N Nobleza, Mariah Siddiqui, Parth V Shah, Prachi Balani, Angel R Lopez, Safeera Khan

**Affiliations:** 1 Neurology, California Institute of Behavioral Neurosciences & Psychology, Fairfield, USA; 2 Neurology, St. George's University, True Blue, GRD; 3 Medicine, California Institute of Behavioral Neurosciences & Psychology, Fairfield, USA; 4 Internal Medicine, California Institute of Behavioral Neurosciences & Psychology, Fairfield, USA; 5 Psychiatry, California Institute of Behavioral Neurosciences & Psychology, Fairfield, USA

**Keywords:** rbd, freezing of gait, parkinson's disease, gait, postural instability, pedunculopontine nucleus, visuoperceptive abnormalities, anticipatory postural adjustment

## Abstract

Rapid eye movement sleep behavior disorder (RBD) contributes to injury due to the alteration of the expected atonia during rapid eye movement (REM) sleep. It occurs before the overt signs of Parkinson's disease (PD). The co-expression of PD and RBD is characterized by non-tremor predominant subtype and higher incidence of freezing. Freezing of gait (FOG) is a debilitating symptom seen in PD patients that lead to falls. While this phenomenon is understood poorly, the involvement of the pedunculopontine nucleus (PPN) and the neural circuits that control locomotion and gait have been examined. This network has also the same control for REM sleep and arousal. The close relationship between PD and RBD and FOG's consequences has led us to explore the relationship between RBD and PD with FOG. This review provides an overview of the neural connections that control gait, locomotion, and REM sleep. The neural changes were seen in PD with FOG and RBD, and sensory and motor changes observed in these two diseases. The functional neuroanatomy that controls REM sleep, arousal, and locomotion overlap significantly with multiple neural circuits affected in RBD and PD with FOG. Visual perception dysfunction and motor symptoms that primarily affect gait initiation are common to both patients with RBD and FOG in PD, leading to freezing episodes. Prospective studies should be conducted to elucidate the relationship of RBD and PD with FOG subtype and find innovative treatment approaches and diagnostic tools for PD with FOG.

## Introduction and background

Rapid eye movement behavior sleep disorder (RBD) is characterized by a loss of normal muscle atonia and subsequent dream enactment [1]. It occurs with other synucleinopathies like Parkinson's disease (PD), multisystem atrophy, and Lewy body dementia in up to 81% of patients from five to 29 years [2]. RBD likely happens before the overt motor, cognitive and autonomic impairments in PD proposed by Braak's staging system, which postulates that medullary structures are affected first in synucleinopathies and eventually ascend to more rostral structures; specifically, a prominent degeneration in the sublaterodorsal nucleus (SLD) will cause rapid eye movement (REM) sleep without atonia and RBD [3, 4]. Polysomnographic studies have shown that 58% of patients with PD have some loss of muscle atonia during REM sleep [5]. This finding is congruent to the study conducted by Sixel-Döring F et al., where they found that 46% of all PD patients had RBD, as diagnosed by polysomnography with video synchronization [6].

Patients with both PD and RBD exhibit a non-tremor-predominant subtype of the disease, and a higher incidence of freezing of gait (FOG), leading to increased frequency of falls [7, 8]. FOG is a debilitating symptom with a complex mechanism understood poorly, occurring in one-third of patients with Parkinson's disease. FOG is defined by an episodic inability for the feet to move forward, despite the intent to walk with predetermined stride length. FOG can have motor phenomena associated with initiation disturbances and transient interruption to stepping while walking. Theories that explain FOG include ineffective gait patterns, alteration in anticipatory postural adjustments (APA), leading to problems in step initiation, perceptual and frontal lobe area dysfunction, disturbed central drive disruptions, and automaticity of gait [9]. While the etiology of FOG is complex, existing evidence states that FOG involves a disruption in neuroanatomical networks in the brainstem, particularly the pedunculopontine nucleus (PPN), a component of the mesencephalic locomotor center postural control circuits responsible for gait and locomotion as well as reward, motivation, compulsion, and REM sleep [10, 11]. Recent work has shown that PPN grey matter atrophy is more evident in patients with idiopathic PD with FOG than those without FOG [12]. These anatomical networks are also involved in RBD's pathophysiology through its thalamic connections to the medial prefrontal and anterior cingulate cortices [10, 13, 14]. It is hypothesized that deficits arising from dysfunction in these regions, such as sleep disorders, are expected to co-express in patients with PD and FOG [8]. The relationship between RBD and Parkinson's disease has been widely explored. Still, only a few published studies elucidate the relationship between RBD and FOG in PD, considering how freezing negatively impacts these patients. This review will provide an overview of the relationship between RBD and FOG by paying attention to the involvement of the PPN and other neural systems responsible for the loss of tone during REM sleep, locomotion, and posture, describe the changes that happen in these neural networks as a result of RBD and PD with FOG and, explore the gait, posture, and sensory and motor abnormalities shared by these two disease entities. 

## Review

Gait, locomotion, and REM sleep control

**Figure 1:**
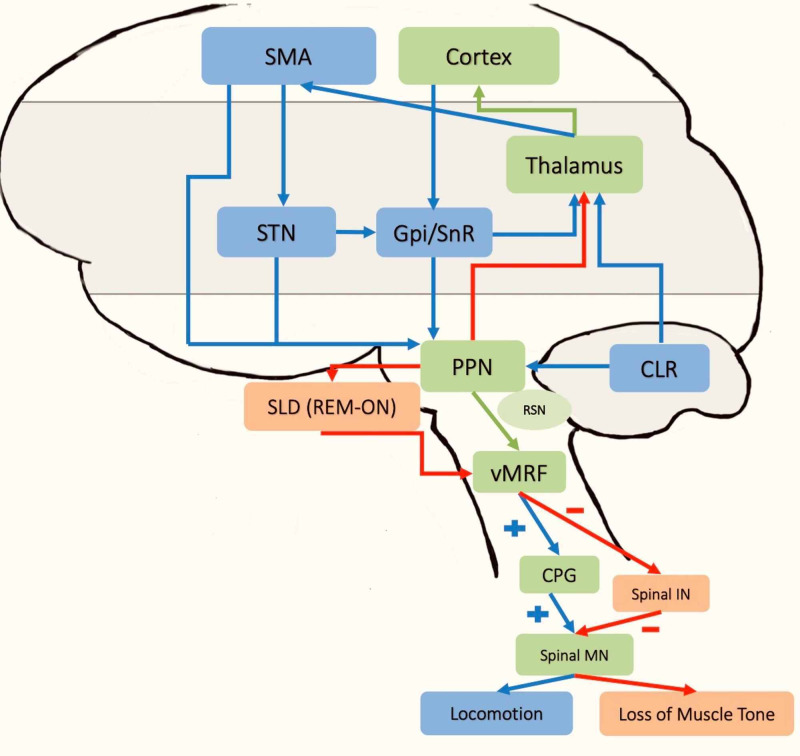
Simplified diagram of the neuroanatomic connections that control arousal, REM sleep, and gait Blue-colored arrows and boxes represent connections exclusive to the motor locomotor region (posture and gait). Red-colored arrows and orange boxes represent the neural network exclusive for REM sleep control. In contrast, green-colored arrows and boxes represent the overlap between the MLR, arousal, and REM sleep control. SMA - supplementary motor cortex; STN - subthalamic nucleus; Gpi/Snr - globus pallidus internus/substantia nigra pars reticulata; PPN - pedunculopontine nucleus; RSN - reticulospinal nucleus; SLD - sublaterodorsal nucleus; vMRF - ventromedial reticular formation; CPG - central pattern generators; CLR - cerebellar locomotor region; Spinal MN - spinal motor neuron, Spinal IN - spinal interneurons; MLR - mesencephalic locomotor region; REM - rapid eye movement

Gait control, locomotion, and REM sleep share common pathways in which the PPN is central to all these functional neuroanatomic connectivities. Figure 1 illustrates the overlap in these structures.

The PPN in the mesopontine tegmentum is involved in locomotor modulation, postural muscle tone, arousal, and REM sleep atonia [15]. Cholinergic cells in the PPN project to the thalamus and increased acetylcholine transmission induces arousal [16]. The same cells have the activity in the REM on and off-center in the brainstem [17]. High-frequency stimulation (>100 Hz) of the PPN results in a suppression of postural muscle tone instead of locomotion [18, 19]. However, a slow stimulation brings the membrane potential firing from a rhythmic state bursting to a state of desynchronized firing observed during arousal or REM sleep [17]. The SLD mediates the loss of muscle tone during REM sleep through its cholinergic connections to the PPN [20]. The PPN is also a part of the mesencephalic locomotor region (MLR) involved in APA and subsequent gait initiation [21]. Acetylcholine stimulation of the caudal PPN (corresponds to the MLR) is associated with suppressing muscle tone via projections to the ventromedial medullary reticular formation (vmRF) while blocking the vmRF with atropine inhibited atonia and facilitated MLR-induced locomotion [22]. With the extensive involvement of the PPN in both REM sleep and locomotion, dysfunction in this area and other neural networks connected to this nucleus can alter REM sleep and poor execution of purposeful movement. The locomotor pathway also includes the reticulospinal nucleus (RSN), which regulates muscle tone. Modulation of RSN during the gait cycle involves both muscle-tone related RSNs and locomotor rhythm. Executing muscle tone regulation during movement requires the integration of all spinal reflex networks. These spinal reflex networks produce rhythm and locomotor movements by activating the central pattern generators (CPG). The CPGs also play a role during the stance phase of the gait cycle. Lastly, the integration of descending supraspinal signals and sensory afferents is necessary for effective control of posture [23]. While CPGs play a role in the gait cycle at the spinal level, purposeful gait requires executive function, attention, and judgment of one's immediate surroundings in which the supraspinal locomotor region (SLR) is involved [24]. This region primarily includes primary and supplementary motor cortices, prefrontal cortices, subcortical structures (basal ganglia, vmRF, MLR, and cerebellar locomotor region) [25]. The subthalamic nucleus (STN), in particular, has an essential role in locomotion control through its direct connection to the supplementary motor area (SMA) and downward projections to the MLR. The STN then serves as a gate to integrate cortical output and cerebellar information by activating or inhibiting the MLR through glutamatergic projections or basal ganglia gamma-aminobutyric acid (GABA)ergic output [26]. The MLR, in turn, relays the information to the central pattern generators (CPGs) [23, 26].

Changes in the neural networks due to freezing of gait in PD and RBD

Neural network changes as a result of FOG in PD and RBD have also been explored. A study by Pozzi et al. determined that the disruption in the communication in the cortical-subthalamic network in the hemisphere with decreased dopaminergic innervation led to gait freezing. This was observed during the transition from a well-executed gait cycle to the onset of gait freezing. Decoupling between the SMA and STN in the hemisphere with decreased dopaminergic innervation suggests asymmetry between the two hemispheres [27]. Asymmetry was also noted in the PPN and its connections to other locomotor regions in a study made by Fling et al., more notably in the right hemisphere frontal cortex and the PPN. Further, lateralization of the PPN tract volume towards the left hemisphere suggests a less accurate and longer time to initiate a task or inhibit an inappropriate action in patients with FOG [28]. Considering these neural networks' asymmetric involvement, will subsequent motor manifestations in these patients also have asymmetric presentations?

Two studies also found a significant reduction in the cerebellar locomotor region (CLR) connection to the PPN in FOG patients [28, 29]. Fasano et al. state that 90% of the lesions in their study were functionally connected to the bilateral dorsal medial cerebellum in FOG patients and a separate anatomic structure that lead to asterixis or hemichorea [29]. These findings show a heterogeneous involvement of the neural networks that control REM sleep, locomotion, and posture. 

Supraspinal control of gait is also affected in RBD and PD with FOG. One study revealed a decrease in PPN functional connectivity with the bilateral SMA proper, pre-SMA, and dentate nucleus in PD with impaired postural instability and RBD group. In particular, the PPN-SMA connectivity defect was seen in PD patients with a positive pull test and prolonged APA duration before gait initiation. PD with impaired postural instability and RBD patients also have decreased PPN functional connectivity with the bilateral medial prefrontal cortex (MPFC). This impaired connectivity is also evident in a study made by Wang et al., where patients with FOG have altered functional connectivity between the PPN and corticopontine-cerebellar pathways and visual temporal areas, including the tracts projecting to motor, sensory and cognitive regions. During tasks requiring high cognitive load, PD patients with FOG had weak activity in the pre-SMA [30]. In contrast, the PD patients without FOG could recruit the prefrontal areas, including the pre-SMA and the medial prefrontal cortex [31]. Simultaneous use of cognition while doing a motor task may lead to ineffective walking and subsequent freezing. 

Patients with RBD were found to have altered functional connectivity between the bilateral PPN and the ventral posterior-anterior cingulate cortex (ACC), regions involved in arousal and alertness. The same structures, known as the functional associative network (ACC and SMA), serve as a compensation network in PD patients with faulty automatic control of sensorimotor networks [30]. However, with RBD and PD co-expression in several patients, this compensatory functional associative network may be rendered useless. Compensatory networks in patients with FOG are also observed in a study by Fling et al. The SMA involved in supraspinal control of gait initiation has increased connection with the MLR and CLR in patients with FOG. In contrast, FOG negative patients had significantly greater functional connectivity than FOG positive patients in the hyper-direct pathway between the STN and SMA. This increased connection implies a reorganization of functional communication within the locomotor network in FOG positive patients. Nevertheless, it fails to serve as a compensatory for freezing, as evidenced by the positive association between FOG severity ratings and increased functional connectivity in the MLR - SMA and CLR - SMA neural networks [32]. 

**Figure 2:**
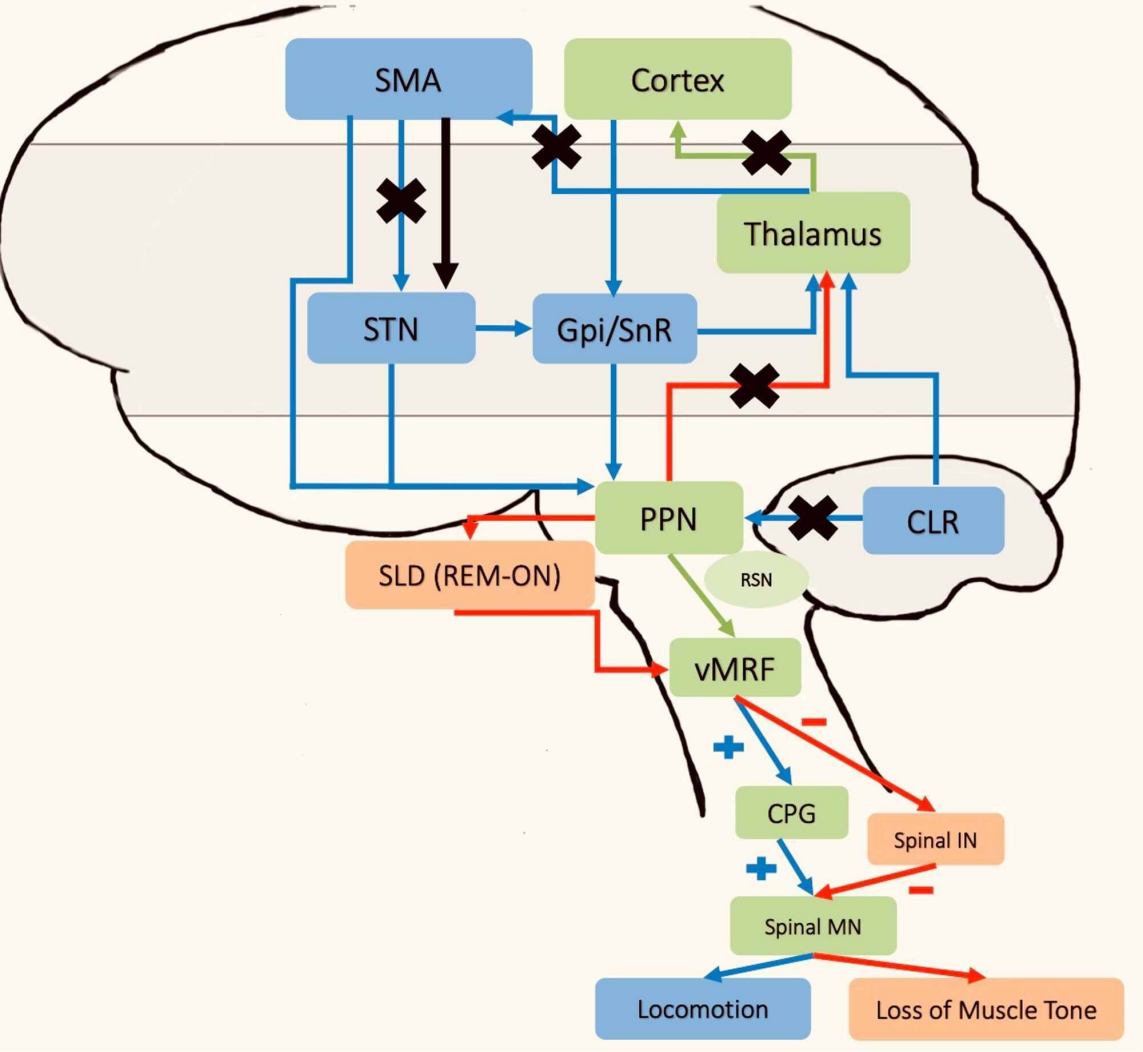
Changes in the neural networks seen in RBD and PD with FOG The cross marks represent the altered connections as a result of RBD and PD in FOG. The black arrow represents the increased connectivity of the SMA to the MLR region, as seen in FOG patients. SMA - supplementary motor cortex; STN - subthalamic nucleus, Gpi/Snr - globus pallidus internus/substancia nigra pars reticulata; PPN - pedunculopontine nucleus; RSN - reticulospinal nucleus; SLD - sublaterodorsal nucleus; vMRF - ventromedial reticular formation; CPG - central pattern generators; CLR - cerebellar locomotor region; Spinal MN - spinal motor neuron; Spinal IN - spinal interneurons; MLR - mesencephalic locomotor region; RBD - rapid eye movement sleep behavior disorder; PD - Parkinson's disease; FOG - freezing of gait

The observed neural changes in these networks do not localize to a single neuroanatomic structure but rather reflect derangements of both spinal and supraspinal networks and, along with it, faulty compensatory mechanisms that worsen freezing. Figure 2 illustrates the changes observed. 

Sensory and motor changes in RBD and FOG

Sensory Changes

The integration of multiple environmental sensory inputs is necessary to execute a purposeful movement. Several studies have looked into these sensory interruptions that contribute to motor impairments in PD and RBD patients. FOG patients unresponsive to levodopa treatment have decreased visuospatial functioning performance as assessed using the Judgment of Line Orientation Test [33]. This visual perception impairment was also tested in FOG and FOG negative PD patients and controls using three sets of doorways with decreasing sizes. Gait velocity was slower in FOG patients during their initial encounter with the doorway. A significant decrease in step length and increased step length variability in FOG patients as they approached the narrow doorway, as well as an increased base of support, were noted [34]. These variables occurring before arrival at the doorway implies that impaired perceptual processes are interrupting the initially planned movement to pass the doorway, suggesting that visuospatial perception, which corresponds to the restructuring of the picture's inherent properties to obtain an overall, meaningful representation, enabling object identification and location determination, contributes to the pathophysiology of gait freezing [34, 35].

Interestingly, idiopathic RBD (iRBD) patients also experience visuoperceptual dysfunction based on poor performance in the Biederman fragmented picture identification task. Results from this test reveal a lack of visual priming, which, when present, generally leads to a faster response to a stimulus as a result of prior exposure. This response seen in iRBD patients is consistent with impaired intermediate perceptive processing of visual information. The initial presentation of an image triggers identifying the overall structural representation of the image [36]. Marques et al. support this finding. In their study, impairment in object identification in RBD using the identification thresholds of incomplete contour drawings of objects is noted, regardless of whether RBD is associated with PD [35]. With the occurrence of visuoperceptive dysfunction in either PD or RBD, it shows that the influence of visuoperceptive dysfunction during purposeful movement is heightened in patients with PD and co-morbid RBD. 

One study also reports auditory and audiovisual perception changes in PD patients. Specifically, there is a significant difference between auditory and visual reaction times in PD, with a more significant alteration observed in the FOG subtype. The responses to visual stimuli were significantly slower compared with the auditory modality [37]. This finding could be explained by the already altered visual perception in these patients, as observed in previous studies. However, it is notable that this difference increases with disease duration and the development of FOG, suggesting a possible compensatory role of the faster auditory reaction time to navigate one's immediate surroundings. Although multisensory facilitation occurs in PD, it is significantly less enhanced than age-matched healthy controls [37]. 

Motor Changes

Motor manifestations in patients with RBD and PD have been described. A study by Postuma et al. has shown that PD with RBD are non-tremor predominant [38], had increased frequency of falls, and had less response to levodopa [31]. Over time, other studies further characterized these findings by examining gait parameters and polysomnographic findings to establish the presence of rigidity and subsequent freezing in patients with PD and RBD. 

Gait parameters were analyzed in various studies. In one study, patients with probable RBD exhibited fewer steps, increased swing time, and step length variability with a tendency to decrease their velocity and cadence [39]. Postural sway as an essential component of the gait cycle was also described by Chen et al., where idiopathic RBD patients showed unrefined sway, which was more evident during difficult situations, as evidenced by increased jerkiness. The noted decrease in sway's smoothness mirrors the nervous system's attempt to correct sway during upright posture. Patients with RBD exhibit decreased smoothness of sway, probably due to rigidity. They also found an increased variability during a forward trunk acceleration, which confers with other study findings [40]. The posterior shift of the center of pressure (CoP) is reduced during the gait cycle's anticipatory and propulsive phase in patients with RBD without coexisting PD [41]. The posterior displacement in CoP is an essential part of anticipatory postural adjustments (APA) to accelerate the body forward and move to the single-stance leg for stabilization before initiation of step [42]. Therefore, failure to shift the CoP leads to gait initiation failure, a higher probability of falls, and freezing. PD with RBD patients and those with RBD alone had longer APA duration [31]. Thevathasan et al. also revealed that patients with PD and FOG had freezing episodes during turning. These patients also needed a more extended time during the turning task. Also, cadence and step length decreased, and variability in step length increased [43]. The similar gait findings seen in both PD with FOG and probable RBD patients support the probability that shared pathophysiology exists between these two disease entities.

Increased muscle activity during REM sleep was found in PD with FOG and RBD patients. The tonic EMG activity, which generally should be low, considering that REM sleep is characterized by loss of muscle tone, increases PD with FOG than in PD without FOG patients [14]. This electromyography (EMG) finding means that REM sleep without atonia (RWSA) occurs more in these patients. RWSA correlates with the disease duration and severity in PD patients as well as RBD based on the RBD screening questionnaire [44]. The increased muscle tone during REM sleep leads to a decreased capacity to generate a posterior shift in the CoP [41]. Also, there is an increased symmetric forearm rigidity in PD with RWSA patients [45]. These findings reflect that RBD's presence in PD patients predisposes them to the FOG subtype, has more gait deficits, and increased rigidity. These findings could also mean that more extensive and earlier neural degeneration in the neural networks that control both REM sleep and locomotion in patients with PD FOG and coexisting RBD leads to a more severe disease presentation. Table 1 presents all the articles reviewed in this study. 

**Table 1:** Summary of all the articles reviewed in this study PD - Parkinson's disease; STN - subthalamic nucleus; FOG - freezing of gait; RCT - randomized controlled trial; CLR - cerebellar locomotor region; DBS - deep brain stimulation; PPN - pedunculopontine nucleus; SMA - supplementary motor area; ACC - anterior cingulate cortex; REM - rapid eye movement; RBD - rapid eye movement sleep behavior disorder; MLR - motor locomotor region; RSWA - rapid eye movement sleep without atonia; EMG - electromyography; APA - anticipatory postural adjustments; iRBD - ﻿idiopathic rapid eye movement sleep behavior disorder

Author and year of publication	Number of patients	Type of study	Results	Conclusion
Pozzi et al. 2019 [27]	Seven PD	Cross-section	﻿A notable decrease in communication between the STN and cortex is evident before transitioning to freezing and recovering from normal walking.	﻿FOG is a result of dysfunction in STN-cortical communication. This finding suggests faulty cortical-subcortical circuitry, which could be a potential area for neuromodulation to improve freezing.
Fasano et al. 2018 [29]	14 PD	RCT	﻿90% of lesions in FOG patients have been located in the CLR, specifically the dorsal medial cerebellum.	This may guide the development of new therapies for treatment-resistant FOG by identifying areas for DBS.
Gallea et al. 2017 [31]	52 PD; 25 controls	Cross-section	﻿As noted in those with sleep disorder and impaired postural control, patients with longer APA have decreased connectivity in the PPN and the SMA and the PPN and ACC, the locomotor, and arousal regions.	﻿The pedunculopontine nucleus and neural networks involved in REM sleep and locomotion support the clinical manifestation and the relationship between RBD and postural control impairments in Parkinson's disease.
Wang et al. 2016 [30]	31 PD 16 controls	Cross-section	﻿FOG patients have decreased PPN and frontal cortex communication compared to FOG negative patients and controls. In particular, the corticopontine-cerebellar pathways are notably affected in addition to the visual temporal areas.	﻿FOG in PD is associated with abnormal PPN and frontal cortex networks, mainly affecting the corticopontine-cerebellar pathways.
Fling et al. 2014 [28]	26 PD patients; 15 age-matched controls	Cross-section	﻿FOG+ patients have increased connectivity between the SMA and MLR and CLR than FOG- patients and controls. This results in increased freezing severity.	﻿There is functional reorganization in the connections between SMA and MLR and the CLR, which leads to a faulty neural compensatory response during FOG.
Fling et al. 2013 [32]	26 PD; patients 15 age-matched controls	Cross-section	﻿There is decreased connectivity of the PPN with the cerebellum, thalamus, and multiple frontal cortex regions, particularly in the right hemisphere. More left hemisphere-lateralized PNN volume was noticed to have poorer performance on cognitive tasks in FOG patients.	﻿FOG is strongly related to neuroanatomic deficits in the right hemisphere's locomotor network.
Thevathasan et al. 2012 [43]	15 PD; nine age-matched controls	RCT	﻿Bilateral PPN stimulation improved gait freezing more than unilateral stimulation but not step length variability.	﻿Bilateral stimulation of a caudal PPN region improves gait freezing but not step length variability.
Fearon et al. 2015 [37]	39 PD; 17 age-matched controls	Cross-section	﻿The PD group had significantly visuoperception ability than healthy controls. Still, auditory reaction times were significantly faster than visual for the PD group only. Multisensory facilitation occurs in PD but is less enhanced than in healthy controls.	﻿﻿There are significant sensory abnormalities in PD. Multisensory abnormalities are not related to disease duration and could be a potential biomarker for the disease.
Factor et al. 2014 [33]	135 PD	Cross Section	﻿The unresponsive FOG group had significantly lower visuospatial ability and executive functioning than other groups. The responsive FOG group was found to exhibit hallucinations.	﻿The unresponsive FOG had executive and visuospatial dysfunction, while responsive FOG is associated with hallucinations suggesting posterior cortical regions' involvement.
Plomhause et al. 2014 [36]	15 iRBD 30 PD 20 age-matched controls	Cross-section	﻿Idiopathic RBD patients lack visual priming effects as assessed by the Biederman task. Parkinson's disease patients with RBD had poorer visuoperceptive performance levels than PD patients without RBD.	﻿There is intermediate visuoperceptive processing dysfunction in idiopathic RBD patients. RBD in PD is associated with decreased visuoperceptive abilities but not attention.
Marques et al. 2009 [35]	20 PD; 10 iRBD; eight age-matched controls	Cross-section	﻿There is poorer performance on object identification seen in PD patients with RBD and idiopathic RBD patients.	﻿This perceptual dysfunction seen in RBD may not be related to the loss of dopamine innervation.
Almeida et al. 2009 [34]	31 PD; 16 age-matched controls	Cross-section	﻿FOG group had step length variability as well as within-trial variability of step length and step time as a response to the narrow doorway.	Freezing may have a perceptual pathology that influences neural movement planning.
Linn-Evans et al. 2020 [45]	61, 41 PD, 20 age-matched controls	Cross-section	Forearm rigidity was significantly higher and more symmetric in the PD-RSWA+ group.	﻿In patients with mild to moderate PD and RSWA, there is associated increased and more symmetric presentation of upper limb rigidity.
Alibiglou et al. 2016 [41]	30 PD	Cross-section	﻿Patients with RBD and PD FOG patients had decreased posterior shift of the center of pressure during gait propulsion. A decreased duration of the initial dorsiflexor muscle burst during gait initiation in both PD and the RBD groups.	﻿People with RBD show alterations in the coupling of posture and gait similar to those seen in PD even before diagnosing a degenerative disorder.
Chahine et al. 2014 [44]	65 PD	Cross-section	﻿Higher amounts of surface EMG activity were associated with longer PD disease duration and greater disease severity.	﻿Surface EMG activity during REM sleep was associated with the severity of both PD and RBD. This EMG result may be useful as a PD biomarker.
Chen et al. 2014 [40]	24 RBD	Cross-section	﻿RBD patients have increased variability of trunk acceleration and decreased smoothness of sway, especially during challenging obstacles. RBD patients demonstrated significant impairment in stereopsis.	Idiopathic RBD patients, particularly those with abnormal stereopsis, have subliminal postural instability under challenging conditions. Postural sway performance can be a biological marker for PD diagnosis.
Videnovic et al. 2013 [14]	30 PD	Cross-section	﻿Tonic muscle activity was increased significantly in the RBD and FOG groups. Phasic muscle activity was significantly increased in the RBD group compared to all other groups.	﻿These findings provide evidence that increased muscle activity during REM sleep is a co-morbid feature of patients with PD with FOG.
McDade et al. 2013 [39]	42 RBD, 492 controls	Cross-section	﻿Probable RBD was associated with decreased velocity, cadence, significantly increased double limb support variability, greater stride time variability, and swing time variability.	﻿Probable RBD is associated with subtle gait changes before PD diagnosis. Diagnosis of probable RBD supplemented by gait analysis may help as a screening tool for the diagnosis of synucleinopathies.
Postuma et al. 2008 [38]	36 PD	Cross-section	﻿Patients with PD and RBD have fewer tremor symptoms. An increased frequency of falls was noted among patients with RBD and are less responsive to their medications.	﻿The presence of altered motor subtypes in PD with RBD suggests a different neurodegeneration pattern compared to PD patients without RBD.

Limitations

This review consisted mostly of cross-sectional studies and a few randomized controlled trials. Longitudinal studies to establish the relationship between RBD and FOG are lacking. In addition, only articles written in English were included in this review; hence, failing to include other studies that may have been relevant to the study.

## Conclusions

This review reveals that the functional neuroanatomy that controls REM sleep, arousal, and locomotion overlap significantly. There is no single neural structure localized that causes both RBD and FOG in PD. Instead, a decreased functionality was identified heterogeneously in these neural networks notably in the cortical-subthalamic network, connections between the PPN and cerebellar locomotor region, PPN and SMA, as well as in the PPN to MPFC and PNN to ACC connectivities. Sensory alterations, particularly visual perception dysfunction is evident in PD and RBD, which predisposes to increased motor symptoms in these patients. These motor symptoms that primarily affect gait initiation are common to both patients with RBD and FOG in PD, which subsequently leads to episodes of freezing. These findings are from cross-sectional studies published to date. Prospective studies are needed to establish all these findings' temporality and better understand the pathophysiology common to RBD and PD FOG. This approach allows better treatment approaches in PD patients with FOG and coexisting RBD and develop early diagnostic tools to diagnose PD with the FOG subtype.
